# Effective Radiative Properties of Tilted Metallic Nanorod Arrays Considering Polarization Coupling

**DOI:** 10.1038/s41598-018-32265-w

**Published:** 2018-09-17

**Authors:** Dustin M. Lattery, Mingeon Kim, Jongin Choi, Bong Jae Lee, Xiaojia Wang

**Affiliations:** 1 Department of Mechanical Engineering, University of Minnesota, Minneapolis, MN 55455 USA; 2 Department of Mechanical Engineering, Korea Advanced Institute of Science and Technology, Daejeon, 34141 South Korea

## Abstract

With the advent of new nanomanufacturing techniques has come the rise of the field of nanophotonics and an increased need to determine optical properties of novel structures. Commercial software packages are able to estimate the behavior, but require large resources and heavy computational time. By combining coordinate transforms and Effective Medium Theory (EMT), an effective relative permittivity tensor is defined and further exploited to calculate the polarization-coupled Fresnel coefficients through Maxwell’s equations. A uniaxial simplification is made to show the case of tilted nanorod arrays. To demonstrate the flexibility of this system, the interfacial reflectance has been calculated for both *s*- and *p*-polarizations as well as the coupled case with the volume filling fractions of *f* = 0.10 and 0.30 for silver (Ag) and titanium (Ti) nanorods, and a scenario of a Ag nanorod array with polymethyl methacrylate (PMMA) as the surrounding medium. The exact results computed by the finite-difference time-domain method justify the validity of EMT with polarization coupling taken into account. The effects of incidence angle and azimuthal angle on reflectance are also discussed. The relatively simple nature of this approach allows for fast estimations of the optical properties of various nanostructures.

## Introduction

Metamaterials with fascinating radiative properties not found in nature have drawn increased attention as of late^1–3^. They have been tested in many applications from increasing energy harvesting efficiency through near-field radiation^4^, radiative cooling^5^, to visual optics that exceed the diffraction limit^6^. They consist of nanoscale three-dimensional periodic assemblies whose properties are determined by the specific size, geometry, material, and orientation of the structures. Arrays consisting of nanorods (NRs) in a dielectric host form simple yet versatile arrangements that show promise for applications involving tunable properties of thermal emission and absorption^4,7,8^. In addition, metallic NRs can also exhibit hyperbolic dispersion in certain wavelengths^9^ and hold promise in both energy conversion^10^ and plasmonic devices^11^. However, the analysis of the radiative properties of these NR array structures is made more complex due to their inherent geometrically induced anisotropy and inhomogeneous nature.

**Figure 1:**
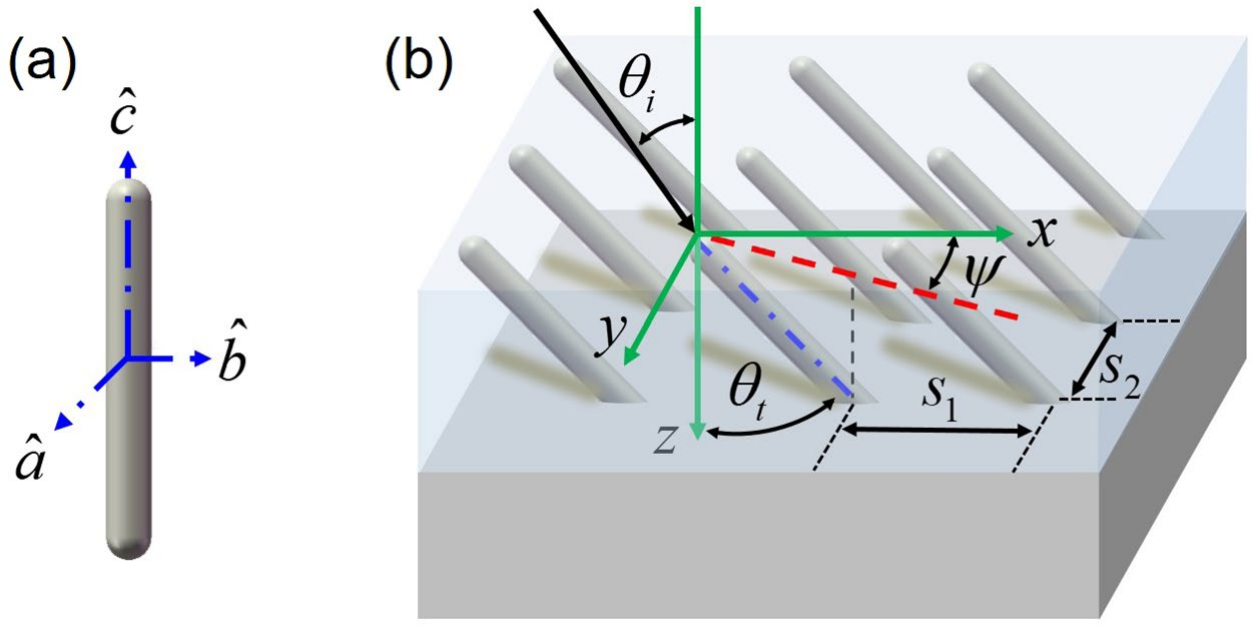
(**a**) Local axes of the single metallic nanorod. The ordinary modes have the electrical field in the *a*-*b* plane while the extraordinary mode has the electric field parallel to the $\hat{c}$ axis. (**b**) Schematic depicting the relationship between the global coordinates (*x*, *y*, and *z*) and the local coordinates defined by the primary optical axis of the NRs ($\hat{c}$). The direction of the incident EM wave is determined by the incidence angle (*θ*_*i*_) and the azimuthal angle (*ψ*). The geometry of the tilted metallic nanorod array is determined by the tilt angle (*θ*_*t*_) and the volume fraction (*f*), which can be found by the spacing between nanorods in the *x* and *y* directions (*s*_1_ and *s*_2_ respectively).

Theoretical methods require the use of estimation techniques such as the effective medium theory (EMT) to describe the field-averaged dielectric function of array-like structures by treating them as effectively homogeneous media. However, due to the geometry-induced anisotropic nature, the effective radiative properties (*i*.*e*., reflectance, transmittance, or absorptance) of such structures are predominantly calculated for samples with specifically controlled angles of incidence and structural orientations (see details in the “Methods” section)^12,13^. In such cases, the polarizations of anisotropic electromagnetic (EM) wave propagation through these complex media are decoupled. For example, incidence of transverse electrical (TE, also *s*-polarization) or transverse magnetic (TM, also *p*-polarization) polarized light will generate only ordinary or extraordinary waves, respectively, in an anisotropic medium, depending on the structural orientation (refer to Fig. 1). To make this EMT-based approach applicable to array-like structures for more universal scenarios, such as *s*- or *p*-polarized incident light exciting both ordinary and extraordinary wave propagation in the array-like structures, a generalized solution is necessary for such polarization-coupled cases.

Herein, we analytically derive a solution to predict the effective radiative properties of array-like anisotropic media that can be extended to the scenarios of polarization coupling. This analytical solution agrees well with finite-difference time-domain (FDTD) simulations when the wavelength is about 10 times greater than the critical dimension of considered structures (which depends on the incident polarization, to be discussed in detail in the “Results” section). The final solution can be readily applied to material systems that contain multiple components and polarization-dependent response to the incident EM waves.

The geometry and related parameters of the array-like structure are depicted in Fig. 1. In the EMT calculation, we conduct a coordinate transform from a local coordinate system (defined by the individual nanorod in Fig. 1a to a global coordinate system in Fig. 1b for the ease of tracing the light propagation. For validation of the EMT calculation, an FDTD simulation is performed to model how the light interacts with the surface of a tilted nanorod system. In the present work, a commercial software, Lumerical FDTD Solutions, is employed. Several studies investigate the optical properties of absorbing nanorod arrays with metallic behaviors^8,13–20^, however, these studies focused on specific conditions with nanorods that are only slightly tilted^14^ or vertically aligned^8,13,15–17^ such that the boundaries of the simulation domain can be simply set as periodic boundary conditions in FDTD. Additionally, several literature studies only analyzed the behavior of the electric field at a very localized area around several nanorods with a finite thickness, which is straightforward to be modeled in an FDTD simulation^18–20^. On the other hand, in the present study, we eliminate the effects of thin-film interference in highly tilted metallic nanorod arrays and actually model the entire nanorod array that extends to the semi-infinite regime using FDTD. Notice that the exact calculation of a semi-infinite metallic nanorod array is crucial for validating the EMT formulation. Details regarding the procedures of EMT and FDTD calculation are provided in the “Methods” section.

## Results

### Decoupled case: wavelength dependence

**Figure 2:**
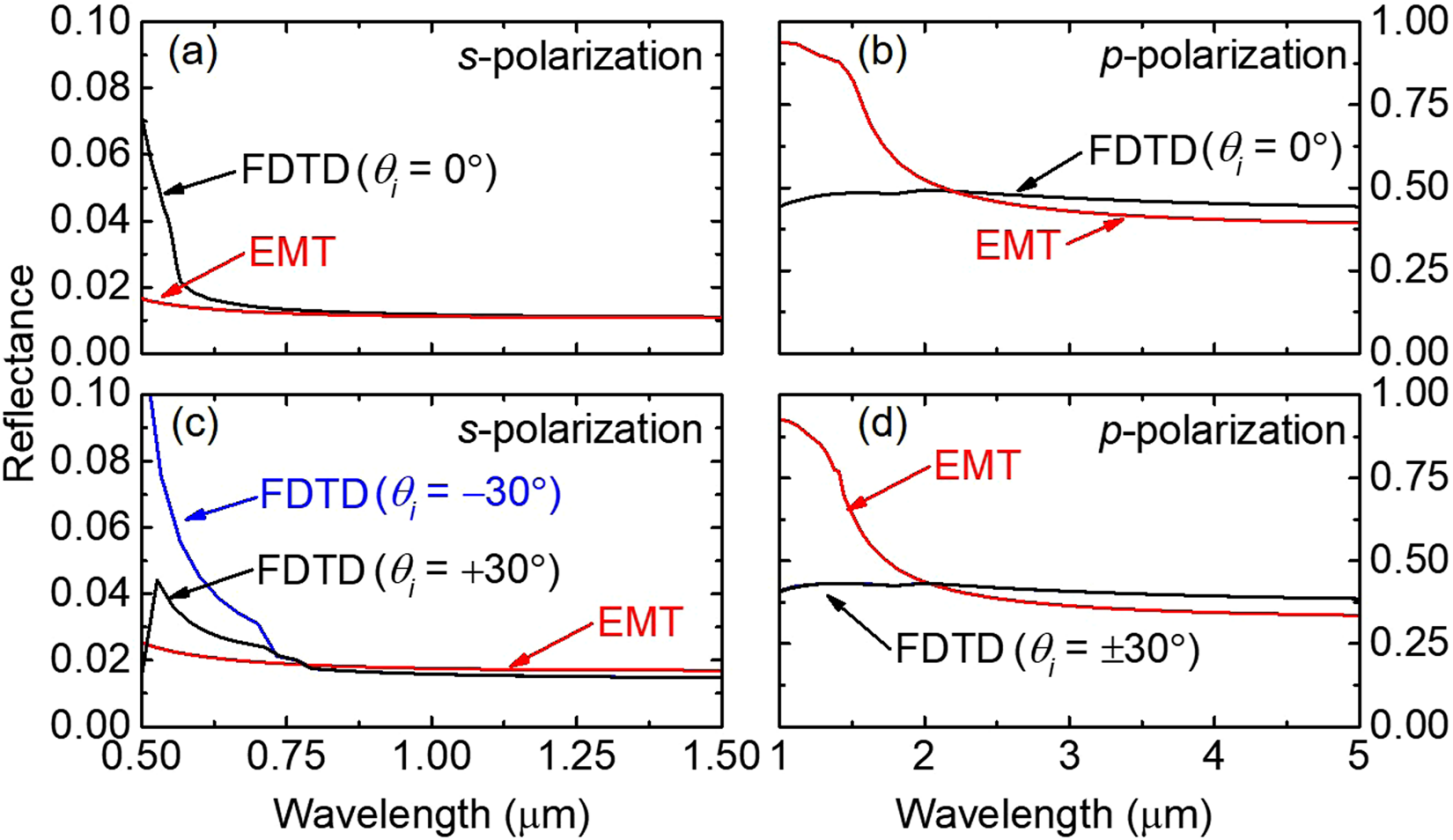
Comparison of the reflectance found from FDTD and EMT as a function of wavelength for Ag nanorods with *D* = 100 nm, *f* = 0.20 (*s*_1_ = 480 nm and *s*_2_ = 280 nm), and *θ*_*t*_ = 73°. The left panel is for the *s* polarization and the right panel is for the *p* polarization.

We have conducted FDTD simulations (see details in the “Methods” section) for a decoupled case (*i*.*e*., the optical axis of the nanorods in the plane of incidence) to justify the validity of our EMT calculation of silver nanorods. In the calculation, the optical constants of silver were taken from the tabulated data in Palik’s handbook^21^. Figure 2 shows the comparison of the predicted reflectance from FDTD and EMT as a function of wavelength for an array of Ag nanorods. It can be clearly seen from Fig. 2 that the EMT generally follows the exact results by FDTD for both polarizations with either normal or oblique incidence (*θ*_*i*_ = ±30°). While the agreement between EMT and FDTD is excellent for long wavelengths, EMT cannot capture the short-wavelength behavior due to the major assumption for EMT validation, which requires that *λ* is ~10 times the characteristic dimension of the material system of interest (as discussed in the “Methods” section). For cases with *s*-polarized light (*i*.*e*., the electric field oscillates parallel to the *y*-axis), EMT begins to agree with FDTD at *λ* ≈ 800 nm for both *θ*_*i*_ = 0° and *θ*_*i*_ = ±30° incidences (Fig. 2c). This is because the electric field in the *s*-polarized case always interacts with a constant nanorod diameter, which does not change with *θ*_*i*_. It is worthwhile to mention that the FDTD results for *θ*_*i*_ = −30° and 30° show different behavior at shorter wavelength region, but become identical at *λ* ≈ 800 nm when the EMT starts to agree with the FDTD. The situation is different for the *p*-polarized case (*i*.*e*., the electric field oscillates in the *x*-*z* plane at an angle of *θ*_*i*_ with respect to the *z*-axis), where the critical wavelength now depends on the projection of the nanorod seen by the electric field. For larger *θ*_*t*_, the wavelength at which the EMT starts to agree with FDTD is much longer than the *s*-polarized case and shifts to slightly smaller wavelengths at oblique incidences. For example, when *θ*_*t*_ = 73°, the comparison between FDTD and EMT suggests a critical dimension of approximately 2000 nm for the *p*-polarized case.

### Coupled case: angular dependence

For polarization-coupled situations, it can be shown that the azimuthal angle *ψ* (refer to Fig. 1) will greatly affect the resulting reflectance. In addition to the *s*- and *p*-polarized reflectance (*R*_*ss*_ and *R*_*pp*_), the cross-polarized reflectances (*i*.*e*., *R*_*sp*_ and *R*_*ps*_ corresponding to the fraction of *s*-polarized incident light that is reflected as *p*-polarized and vice versa) must be accounted for. These terms can be calculated from EMT through the Fresnel coefficients *r*_*sp*_ and *r*_*ps*_ (see Supplementary Information); however, determining the coupled reflectivity through the FDTD simulation is a non-trivial task (see “Methods” section for details).

**Figure 3:**
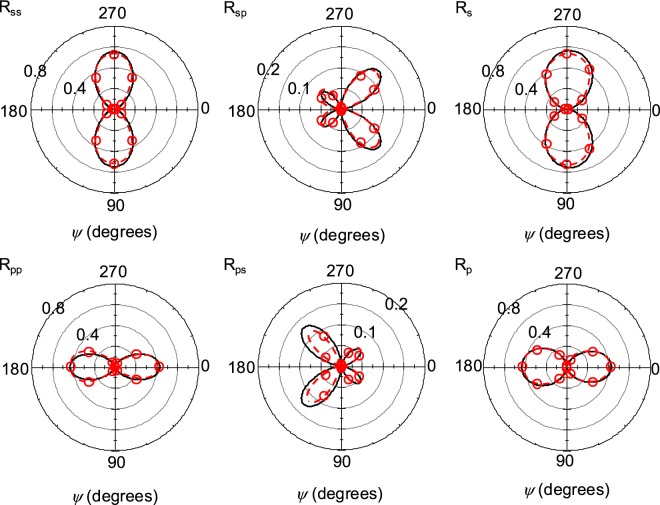
Coupled reflectance from an Ag nanorod structure (*D* = 100 nm, *f* = 0.20, *s*_1_ = 480 nm, *s*_2_ = 280 nm, and *θ*_*t*_ = 73°) at *λ* = 2000 nm and *θ*_*i*_ = 30°. Results from EMT are shown as black lines and FDTD results are shown as red circles with a dashed red line to guide the eye. *R*_*s*_ (or *R*_*p*_) indicates the total reflectance from incident *s*-polarized (or *p*-polarized) light; that is, *R*_*s*_ = *R*_*ss*_ + *R*_*sp*_ (or *R*_*p*_ = *R*_*pp*_ + *R*_*ps*_).

Figure 3 shows the dependence of *ψ* on the polarization-resolved reflectance for the oblique incidence of *θ*_*i*_ = 30° and *λ* = 2000 nm, where the EMT agrees well with FDTD for both polarizations. When *ψ* = 0°, the two polarizations are decoupled, and the resulting *R*_*ss*_ is smaller than *R*_*pp*_. Rotating the nanorods to *ψ* = 90° provides the opposite behavior (larger *R*_*ss*_ than *R*_*pp*_), which is captured by both the trends of FDTD and EMT. When the polarization coupling effect appears (*i*.*e*., *ψ* ≠ 0), the EMT calculation agrees excellently with the FDTD simulation. Similarly to the relation between *R*_*ss*_ and *R*_*pp*_, *R*_*sp*_ and *R*_*ps*_ exhibit similar azimuthal angle dependence; that is, *R*_*sp*_ at *ψ* corresponds to *R*_*ps*_ at *ψ* + 90°.

### Parametric study

Figures 2 and 3 clearly verify that our EMT formulation can successfully predict the radiative properties of tilted nanorod arrays by considering polarization coupling. In order to better understand the impact of the interface reflectance from nanorod arrays, a parametric study has been conducted using the EMT to show the impact of the filling fraction, the nanorod material, the material of surrounding medium, and the azimuthal angle. Notice that the geometric parameters of nanorod arrays, such as the diameter, *s*_1_, and *s*_2_, are not considered hereafter because the EMT is proven to be valid in the chosen wavelength region (1.5–3 *μ*m).

#### Filling fraction

**Figure 4:**
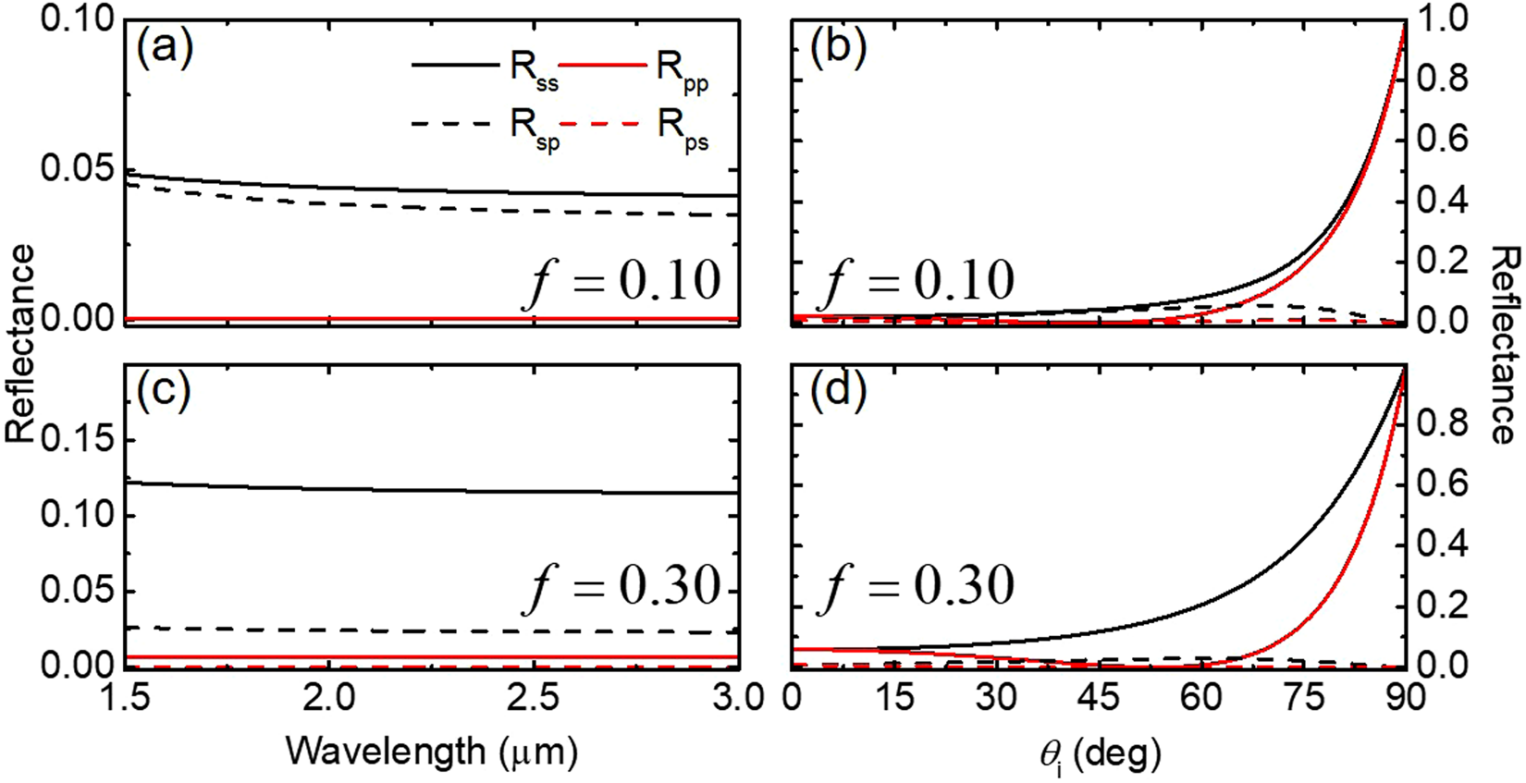
Spectral and angular reflectance at the interface between air and an Ag nanorod array with varying filling fraction. (**a**) and (**c**) show the spectral reflectance from 1.5 to 3.0 *μ*m for *f* = 0.10 and *f* = 0.30, respectively, and *θ*_*i*_ = 45°, *θ*_*t*_ = 45°, and *ψ* = 45°. (**b**) and (**d**) show reflectance as a function of incidence angle *θ*_*i*_, with *θ*_*t*_ = 45°, *ψ* = 45°, and *λ* = 2000 nm for *f* = 0.10 and 0.30, respectively.

One of the most critical parameters to understand for nanorod arrays is the filling fraction. Small inter-wire spacing often leads to enhanced radiation transport through surface plasmons, which are thought to be the reason for enhanced Raman signals seen by silver nanorods^22^. Figure 4 shows the coupled calculation at the interface between air and Ag NRs with *f* = 0.1 and 0.3. Although Ag is highly reflective in the near-infrared (NIR) spectral region, the Ag NR array with *f* = 0.1 shows greatly suppressed reflection (total reflectance is about 0.1), as shown in Fig. 4a. Interestingly, a large cross-polarization term (*R*_*sp*_) exists in the considered spectral region. This term is largely dependent on both the magnitude of the single polarization terms and the azimuthal angle. Increasing the filling fraction results in an increase in *s*- and *p*-polarized reflectance over the NIR region, but a decrease in the magnitude of the cross-polarization terms (see Fig. 4c). For the dependence of reflectance on the incident angle depicted in Fig. 4b and d, *R*_*ss*_ and *R*_*sp*_ follow the typical trend of polarized light incident on a semi-infinite medium; that is, *R*_*ss*_ increases monotonically with *θ*_*i*_, while *R*_*pp*_ exhibits a minimum at a pseudo-Brewster angle. An increase in the volume fraction shifts the Brewster angle to a larger *θ*_*i*_. The cross-polarization terms remain relatively small for the entire range of *θ*_*i*_.

#### Impact of nanorod materials

**Figure 5:**
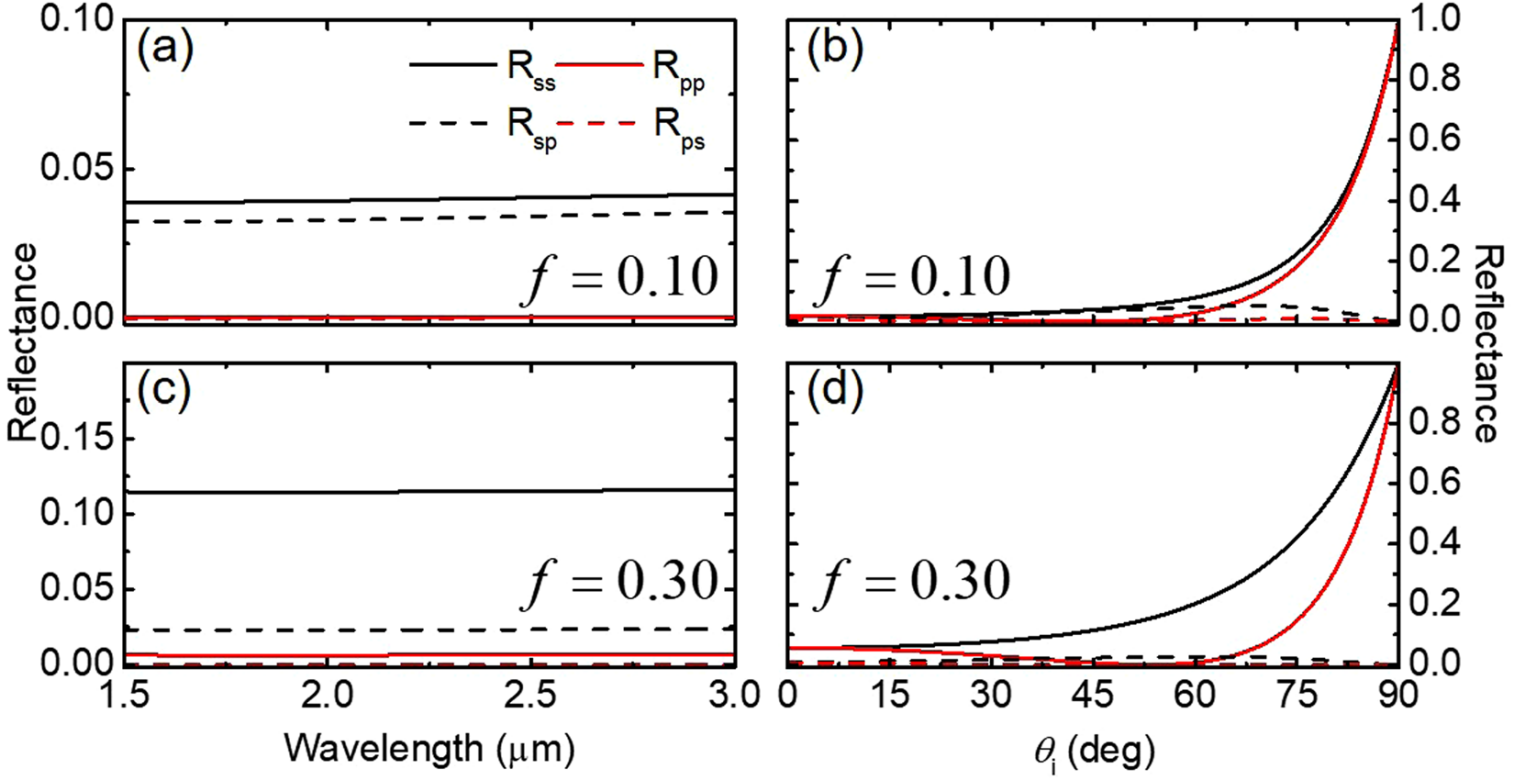
Spectral and angular reflectance at the interface between air and a Ti nanorod array with varying filling fraction. (**a**) and (**c**) show the spectral reflectance from 1.5 to 3.0 *μ*m for *f* = 0.10 and *f* = 0.30, respectively, and *θ*_*i*_ = 45°, *θ*_*t*_ = 45°, and *ψ* = 45°. (**b**) and (**d**) show reflectance as a function of incidence angle *θ*_*i*_, with *θ*_*t*_ = 45°, *ψ* = 45°, and *λ* = 2000 nm for *f* = 0.10 and 0.30, respectively.

While Ag nanorods are one of the most prevalent tilted nanorod systems, glancing angle deposition (dynamic shadowing growth) is able to create nanorod arrays from a variety of materials, including metals, semiconductors, and various metal-oxides^23^. Our proposed EMT approach considering the polarization coupling effect can be readily applied for such versatile systems with a great flexibility and ease of computation. Figure 5 shows the reflectance of an array of titanium (Ti) nanorods as a function of wavelength and incidence angle for two filling fractions of *f* = 0.1 and 0.3, respectively. As shown by Fig. 5a, the TiNRs exhibit extremely low reflectance (less than 10% of incident intensity is reflected). Since the nanorods are assumed to be semi-infinite (*i*.*e*., their length is much longer than the optical penetration depth), the absorptance of TiNRs would approach the optical properties of vertically aligned carbon nanotubes^23^. Similar to AgNRs, an increase of the filling fraction from 0.1 to 0.3 will increase *s*− and *p*−polarized reflectance while decreasing the cross-polarization terms of *R*_*sp*_ and *R*_*ps*_ (Fig. 5c). Comparing TiNRs to AgNRs in the same conditions (Fig. 4), the reflectance of TiNRs is nearly constant as opposed to decreasing when the wavelength increases (such as in AgNR). This difference is because Ti is lossier (*i*.*e*., the imaginary part of its dielectric function is greater) than Ag in the considered wavelength region^24^.

#### Surrounding medium materials

**Figure 6:**
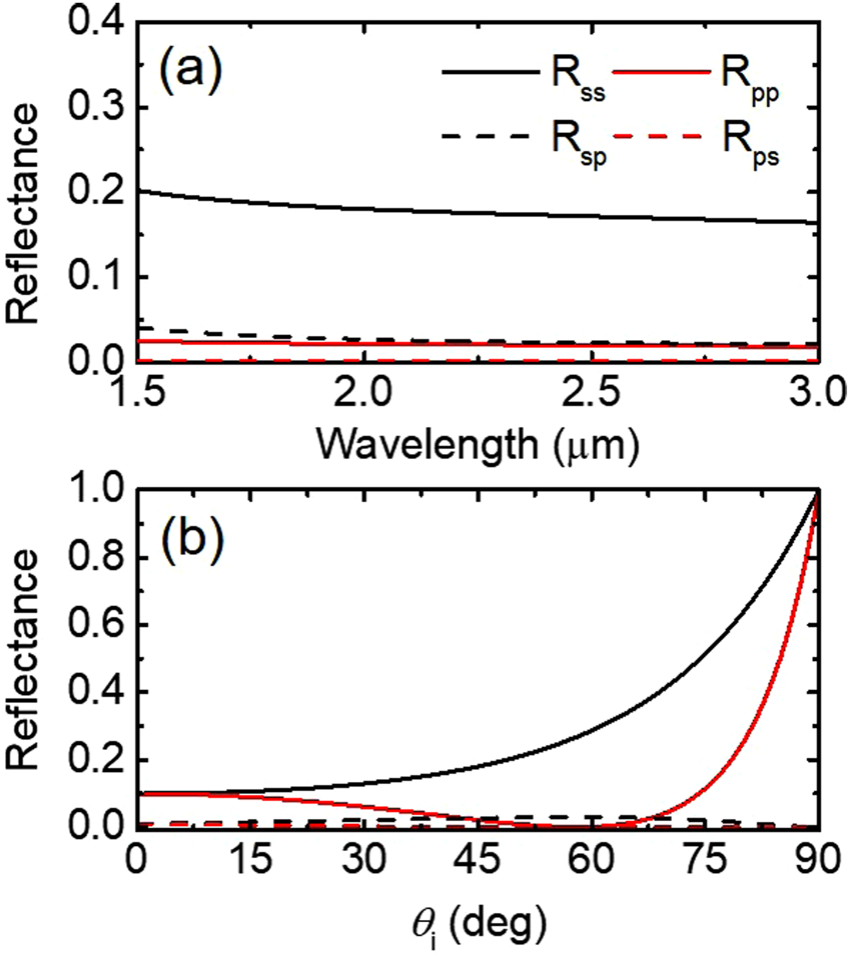
Spectral and angular reflectance of an optically opaque array of Ag nanorods with a surrounding medium of PMMA. Calculation parameters are (**a**) *f* = 0.10, *θ*_*i*_ = 45°, *θ*_*t*_ = 45°, and *ψ* = 45°; (**b**) *f* = 0.10, *θ*_*t*_ = 45°, *ψ* = 45°, and *λ* = 2000 nm.

In all of the previously discussed simulations, the material surrounding the nanorods is treated as air, but taking this term to be non-unity (*n* ≠ 1) will result in noticeable changes to the optical properties of the interface between the incident medium and the array structure. Figure 6a is produced using the same parameters as Fig. 4, but with the addition of polymethyl methacrylate (PMMA) as the surrounding medium. PMMA is chosen as a representative surrounding material in this work, based on the following reasons: (1) optical polymers, including PMMA, generally have dielectric behaviors with similar indices of refraction (n  ≈  1.4–1.5 and *κ* = 0) that are relatively wavelength independent over the spectral regime of interest in this work^25^; and (2) spin coating of PMMA is a common practice that can be easily applied as the host medium for nanorod array structure. Comparison of Figs 4a and 6a suggests that the refractive index of the surrounding medium greatly affects the *R*_*ss*_ and *R*_*pp*_ such that *s*- and *p*-polarized reflectance of the metallic nanorods in PMMA are higher than those of the case in air. However, the cross-polarization terms, *R*_*sp*_ and *R*_*ps*_ exhibit little variation with respect to the change of the surrounding medium, suggesting that cross-polarized reflectance may mainly be induced by the geometrical effect of the tilted nanorod array. Figure 6b also shows a behavior in that *p*-polarization term approaches zero at a pseudo-Brewster angle which results in an even greater ratio of the reflected *s*-polarized light to any other polarization.

#### Azimuthal angle

**Figure 7:**
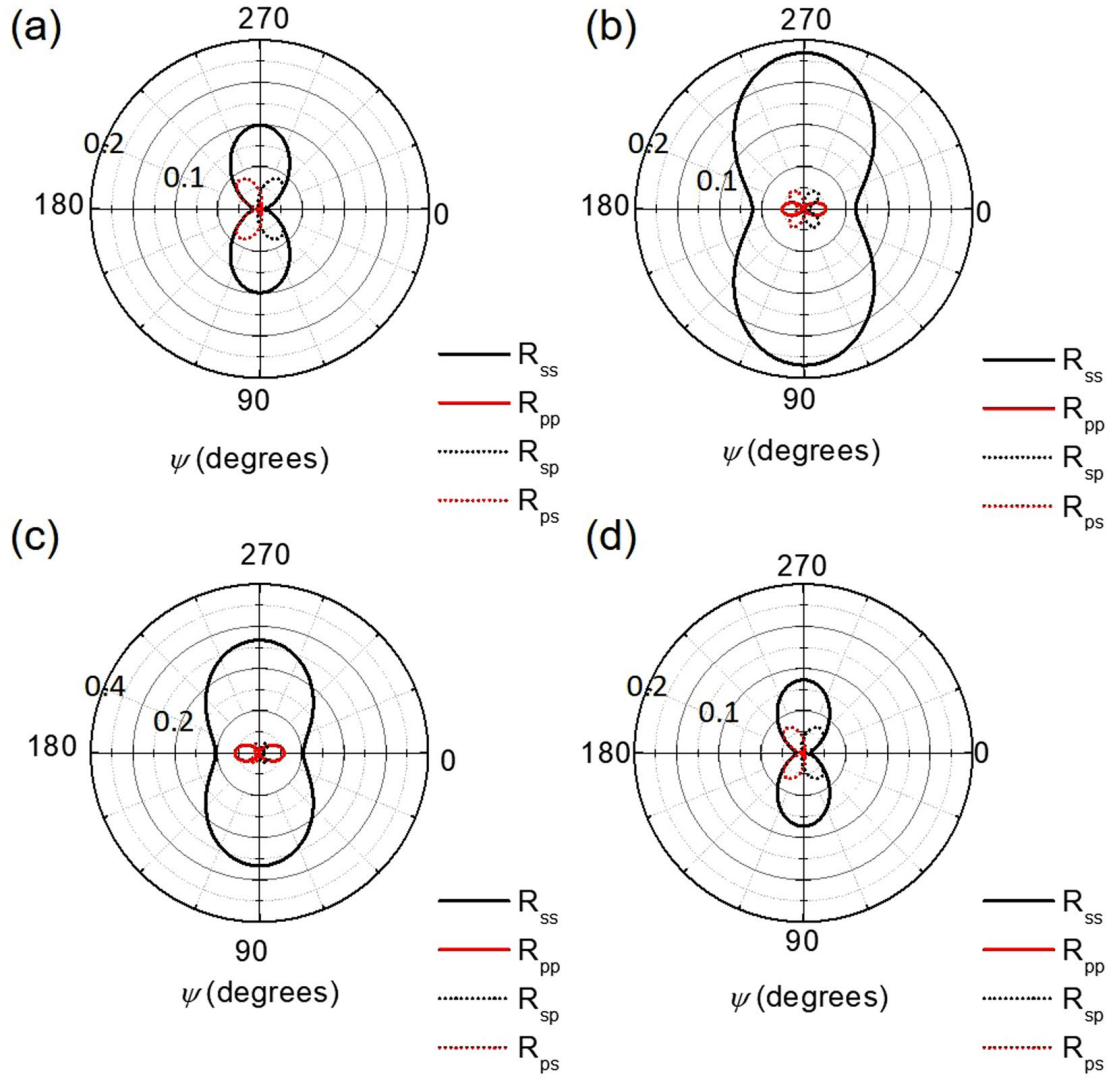
Reflectance as a function of azimuthal angle *ψ* at *λ* = 2000 nm. (**a**) and (**b**) show the behavior of varying Ag filling fractions, *f* = 0.10 and 0.30 respectively. (**c**) shows the behavior in the case of Ag nanorods (*f* = 0.10) in a surrounding medium of PMMA. (**d**) plots the calculation results for an array of Ti nanorods in air with a filling fraction of *f* = 0.10.

All coupled derivations include the azimuthal angle, which is the main difference between coupled and decoupled models. This term leads to cross polarizations that greatly complicate the predicted reflectance, but once the model has been made, the impact of azimuthal rotation can be plotted, as illustrated in Fig. 7. In the decoupled limit (*ψ* = 0°), the cross-polarization terms go to zero, which is another verification of the robustness of this model. Due to the nature of the coordinate system transform, the cross-polarization terms are equal, but seen at angles 180° offset from each other. This behavior could be used to determine the direction of the nanorod array simply by looking at the magnitude of cross-polarization terms. While these terms are typically small (as shown in Fig. 7) compared to the *R*_*ss*_ and *R*_*pp*_ terms, there are conditions when they can be large, such as in Fig. 6, where the *R*_*sp*_ dominates over *R*_*pp*_ in a small wavelength window. The magnitudes of these cross-polarization terms are always maximized at *ψ* = 45°, 135°, 225°, and 315°.

## Conclusion

In this work, we investigate the polarization-coupled radiative properties of metallic nanorod arrays by combining EMT and anisotropic wave propagation. The Maxwell-Garnett EMT is utilized to obtain ordinary and extraordinary relative permittivities. A coordinate transform of the local permittivity results in a global permittivity tensor that can be used to calculate the Fresnel coefficients. The exact results computed by FDTD simulation justify the validity of EMT with polarization coupling taken into account.

Parametric studies have also been conducted to examine the effects of wavelength, filling fraction, incidence angle, nanorod material, and azimuthal angle. Increasing the filling fraction tends to enhance the *s*- and *p*-polarized reflectance while decreasing the cross-polarized reflectances (*i*.*e*., *R*_*sp*_ and *R*_*ps*_). Changing the nanorod material from silver to titanium can greatly change the optical behavior, even at low filling fraction, resulting in a much more absorbing material that is also less dependent on incidence angle. The final study showed that changing the surrounding medium to a polymer infill will increase the impedance mismatch at the air/array interface and thus increase *R*_*ss*_ and *R*_*pp*_ of the array structure, while keeping *R*_*sp*_ and *R*_*ps*_ relatively small. This indicates that the geometry of the nanorod array has a large impact on the cross-polarized reflectance.

The theoretical simulation results presented point to the potential of metallic nanorod arrays as optical gratings, Brewster windows, and other optical instruments. Further work is needed to explore a larger range of nanorod materials and geometries and to better understand the light path in the event of multiple interfaces as seen in thin-film optics. Because this is a single interface with an effective medium assumption, it is a relatively simple calculation compared to other simulation methods (*e*.*g*., FDTD in this work) that are computationally expensive while capable of simulating more complicated material structures.

## Methods

### Effective medium theory

Analytical derivation of the reflectance of small embedded particles requires the consideration of multiple reflections within the structure. The EMT has been widely used to approximate the effective properties of a medium consisting of two or more components. Two main approximations have been widely used to predict the effective permittivity based on volume-averaged fields. In the case of two component materials, both the Maxwell-Garnett (MG)^26^ approximation and the Bruggeman^27^ approximation consider a filler material (a) embedded in a host material (b) and compute an effective relative permittivity as a function of the relative permittivity of each material, a volumetric filling fraction (*f*), and a depolarization factor (*g*). The MG approximation is given as

1 $${\varepsilon }_{eff}={\varepsilon }_{b}+{\varepsilon }_{b}\frac{f({\varepsilon }_{a}-{\varepsilon }_{b})}{g(1-f)({\varepsilon }_{a}-{\varepsilon }_{b})+{\varepsilon }_{b}}$$

and is used typically for low filler concentration (*f* < 30%). In Eq. (1), *g* is the depolarization factor that is shape-specific for the filler materials dispersed as particles in the host media. The depolarization factor of filler particles with random shape can be rather complicated to calculate. However, for the case of NRs which can be treated as a prolonged ellipse with a uniaxial geometry (cigar-like, Fig. 1a), two of the axes along the radial directions are equivalent, degenerating to the “ordinary” state leaving the third axis along the axial direction being the “primary extraordinary” state (also called the optical axis of the uniaxial medium to be discussed later). This simplification gives two depolarization factors corresponding to “primary extraordinary” and “ordinary”, respectively:

2 $${g}_{{\rm{E}}}=\frac{1-{e}^{2}}{{e}^{2}}[\frac{1}{2e}\,\mathrm{ln}(\frac{1+e}{1-e})-\,1]\,{\rm{and}}\,{g}_{{\rm{O}}}=\frac{1}{2}(1-{g}_{{\rm{E}}})$$

Both *g*_E_ and *g*_O_ depend on the eccentricity of the prolonged ellipsoidal NR:

3 $$e=\sqrt{1-{(D/L)}^{2}}$$

where *D* and *L* correspond respectively to the diameter and length of the NR. The degeneration of *g* results in two distinctive effective permittivities for a NR: the “ordinary permittivity” (*ε*_O_) along the radial direction calculated by using *g*_O_ and the “extraordinary permittivity” (*ε*_E_) along the axial direction calculated by using *g*_E_.

For spherical particles with *ε*_E_ ≈ *ε*_O_, the system behaves as an effectively isotropic material. Whereas for aligned NRs, the difference in *ε*_E_ and *ε*_O_ results in an effective uniaxial medium consisting of NRs and air. This uniaxial medium has a single optical axis parallel to the axial direction of the aligned NRs as denoted by in Fig. 1. This shape-induced uniaxial feature leads to anisotropic wave propagation in the aligned NRs that is highly dependent on the orientation of the optical axis and the direction of incident light.

For reasonable predictions on the effective radiative properties of aligned NR array using the EMT, it is also important that the characteristic dimension of the structure should be much less than the wavelength of the incident light. In this case, the incident EM wave does not see individual NRs, but rather, it interacts with an effectively homogeneous medium with properties averaged over all components. The application for EMT typically requires that *λ* is at least 10 times longer than the critical dimension. Because of the ease of application, these theories are sometimes used for parameter extraction in situations that do not meet the characteristic dimension requirement, as discussed by Smith *et al*.^28^. However, EMT can provide a first estimation of the optical properties even for cases like these.

Using previously discussed formulas^29^, this permittivity tensor can be used to calculate Fresnel coefficients, including: *r*_*ss*_ (the Fresnel coefficient corresponding to *s*-polarized light that is reflected as *s*-polarized light), *r*_*sp*_ (*s*-polarized light that is reflected as *p*-polarized light), *r*_*ps*_ (*p*-polarized light that is reflected as *s*-polarized light), and *r*_*pp*_ (*p*-polarized light that is reflected as *p*-polarized light).

Note that for isotropic cases, or cases where the optical axis is inside the plane of incidence (*ψ* = 0), the cross-polarization terms (*r*_*sp*_ and *r*_*ps*_) will reduce to zero. In these special situations, there is no coupling between electric fields for the ordinary and extraordinary cases and they are thus are decoupled cases. For these decoupled cases, *β* = 0, which causes the permittivity tensor to simplify to an admittance and impedance approach as described by Gaylord *et al*.^30^, resulting in *r*_*ss*_ and *r*_*pp*_ being equivalent to *r*_12*s*_ and *r*_12*p*_ as defined by Wang *et al*.^12^.

### Finite-difference time-domain simulation

In order to calculate the reflectance of arrays of tilted nanorods, a three-dimensional simulation domain with a mesh of 5-nm cubes was created, and periodic boundary conditions in the *x*- and *y*-direction, and a perfect matching layer (PML) in the *z*-direction were set. The auto shutoff ratio is set to be 10^−6^. In general, a semi-infinite medium can be approximated by a thin (*i*.*e*., finite thickness) opaque layer. Since the metallic nanorod array can be either highly absorbing or highly transparent, depending on the polarization state, we cannot make it opaque for both polarizations. Furthermore, the FDTD simulation will be unstable when a metallic structure penetrates or overlaps the PML^31^. Therefore, a special trick is applied to simulate the reflectance of a semi-infinite metallic nanorod array. In the simulation, we actually employ a free standing metallic nanorod array with the finite thickness of 30 *μ*m, which is much greater than the wavelength of incident light. Therefore, there must be reflected waves from the bottom interface between the nanorod array and air. To avoid collecting the reflected energy from the bottom interface, we adjust the simulation time so that the calculation is finished before the reflected beam from the bottom interface arrives at the monitor. In our simulation, the time that the wave was initially emitted by the source initially reaches the top surface of the nanorod array and the time that the reflected wave from the top surface of the nanorod array reaches the monitor is 2 fs. An additional 20 fs is also required for the fluctuation of the light source to be stabilized, which means that at least 22 fs is required to collect the reflected beam from the top surface of the nanorod array. To assure numerical convergence, we set the system to allow a total simulation time of 200 fs (*i*.*e*., about 10 times the minimum required time). To satisfy this criterion, the thickness of the tilted nanorod array should be at least 30 *μ*m so that the reflected light from the bottom interface cannot reach the monitor before 200 fs. Although it is not shown here, the reflectance of a semi-infinite material with a refractive index of 1.4 is calculated by the aforementioned trick and compared with the analytical solution using the Fresnel coefficient. The relative error of the FDTD simulation is found to be less than 1%.

Regarding the coupled case, since the Fresnel coefficient is defined as the electric field of the reflected light relative to the electric field of the incident light, in order to calculate the cross-polarization terms (*r*_*sp*_ and *r*_*ps*_) the electric field of the reflected light must be analyzed in the coordinate system of the incident light. Therefore, in order to calculate the cross-polarization terms, the electric field detected at the monitor in the FDTD coordinate system should converted into the coordinate system of the incident light. Since the incident light has both the azimuthal angle *ψ* and the incidence angle *θ*_*i*_, we extract the *x*-, *y*-, and *z*-components of the electric field, and use the Euler angle transform to convert the coordinate system. It can be seen that after the transformation is performed, the electric field of the reflected light is represented by only two components of *s*- and *p*-polarization on the incident light coordinate system. The cross-polarization terms were calculated through the ratio of the polarized reflected electric field to that of the polarized incidence. By taking average of the Fresnel reflection coefficient over the unit cell of the nanorod array in the near field, one can estimate the reflectance^32^.

## Electronic supplementary material

Supplementary Information

## References

[1] Liu Y, Zhang X (2011). Metamaterials: a new frontier of science and technology. Chemical Society Reviews.

[2] Pawlik G, Tarnowski K, Walasik W, Mitus AC, Khoo IC (2014). Liquid crystal hyperbolic metamaterial for wide-angle negative–positive refraction and reflection. Optics Letters.

[3] Wang B-X, Zhai X, Wang G-Z, Huang W-Q, Wang L-L (2015). Frequency tunable metamaterial absorber at deep-subwavelength scale. Optical Materials Express.

[4] Chang J-Y, Basu S, Wang L (2015). Indium tin oxide nanowires as hyperbolic metamaterials for near-field radiative heat transfer. Journal of Applied Physics.

[5] Raman AP, Anoma MA, Zhu L, Rephaeli E, Fan S (2014). Passive radiative cooling below ambient air temperature under direct sunlight. Nature.

[6] Zhang X, Liu Z (2008). Superlenses to overcome the diffraction limit. Nature Materials.

[7] Chang J-Y, Yang Y, Wang L (2015). Tungsten nanowire based hyperbolic metamaterial emitters for near-field thermophotovoltaic applications. International Journal of Heat and Mass Transfer.

[8] Bao H, Ruan X, Fisher TS (2010). Optical properties of ordered vertical arrays of multi-walled carbon nanotubes from FDTD simulations. Optics Express.

[9] Poddubny A, Iorsh I, Belov P, Kivshar Y (2013). Hyperbolic metamaterials. Nature Photonics.

[10] Wang Y (2012). Metamaterial-plasmonic absorber structure for high efficiency amorphous silicon solar cells. Nano Letters.

[11] Kabashin AV (2009). Plasmonic nanorod metamaterials for biosensing. Nature Materials.

[12] Wang XJ, Abell JL, Zhao Y-P, Zhang ZM (2012). Angle-resolved reflectance of obliquely aligned silver nanorods. Applied Optics.

[13] Wang H, Liu X, Wang L, Zhang Z (2013). Anisotropic optical properties of silicon nanowire arrays based on the effective medium approximation. International Journal of Thermal Sciences.

[14] Bao H, Duvvuri B, Lou M, Ruan X (2014). Effects of randomness and inclination on the optical properties of multi-walled carbon nanotube arrays. Journal of Quantitative Spectroscopy and Radiative Transfer.

[15] Bao H, Ruan X (2010). Optical absorption enhancement in disordered vertical silicon nanowire arrays for photovoltaic applications. Optics Letters.

[16] Cansizoglu H, Cansizoglu MF, Finckenor M, Karabacak T (2013). Optical absorption properties of semiconducting nanostructures with different shapes. Advanced Optical Materials.

[17] Patchett S, Khorasaninejad M, Nixon O, Saini SS (2013). Effective index approximation for ordered silicon nanowire arrays. Journal of Optical Society of America B.

[18] Liu Y-J, Zhang Z-Y, Zhao Q, Dluhy RA, Zhao Y-P (2009). Surface enhanced raman scattering from an Ag nanorod array substrate: the site dependent enhancement and layer absorbance effect. Journal of Physical Chemistry C.

[19] Gao R (2018). SERS polarization-dependent effects for an ordered 3D plasmonic tilted silver nanorod array. Nanoscale.

[20] Kim M (2018). Accordion-like plasmonic silver nanorod array exhibiting multiple electromagnetic responses. NPG Asia Materials.

[21] Smith, D., Shiles, E., Inokuti, M. & Palik, E. *Handbook of optical constants of solids* (Academic Press, Inc. Orlando, 1998).

[22] Oh M-K (2015). Morphological and SERS properties of silver nanorod array films fabricated by oblique thermal evaporation at various substrate temperatures. Nanoscale Research Letters.

[23] Zhao Y (2014). Dynamic shadowing growth and its energy applications. Frontiers in Energy Research.

[24] Wang XJ, Flicker JD, Lee BJ, Ready WJ, Zhang ZM (2009). Visible and near-infrared radiative properties of vertically aligned multi-walled carbon nanotubes. Nanotechnology.

[25] Sultanova N, Kasarova S, Nikolov I (2009). Dispersion proper ties of optical polymers. Acta Physica Pol. A.

[26] Maxwell GJ (1904). Colours in metal glasses and metal films. Philos. Trans. R. Soc. London, Sect. A.

[27] Bruggeman D (1935). The calculation of various physical constants of heterogeneous substances. I. the dielectric constants and conductivities of mixtures composed of isotropic substances. Ann. Phys..

[28] Smith DR, Vier DC, Koschny T, Soukoulis CM (2005). Electromagnetic parameter retrieval from inhomogeneous metamaterials. Physical Review E.

[29] Lekner J (1991). Reflection and refraction by uniaxial crystals. Journal of Physics: Condensed Matter.

[30] Weis RS, Gaylord TK (1987). Electromagnetic transmission and reflection characteristics of anisotropic multilayered structures. Journal of the Optical Society of America A.

[31] Deinega A, Valuev I (2011). Long-time behavior of PML absorbing boundaries for layered periodic structures. Computer Physics Communications.

[32] Lee BJ, Chen Y-B, Zhang ZM (2008). Confinement of infrared radiation to nanometer scales through metallic slit arrays. Journal of Quantitative Spectroscopy and Radiative Transfer.

