# Supplementary material for: Effective Radiative Properties of Tilted Metallic Nanorod Arrays Considering Polarization Coupling
Source: Sci Rep. 2018 Sep 17;8:13896. doi: 10.1038/s41598-018-32265-w (PMC6141545; doi:10.1038/s41598-018-32265-w)
Supplement: Supplementary file 1 — Supplementary Information [file 41598_2018_32265_MOESM1_ESM.pdf]

Supplementary Information: Effective Radiative Properties of Tilted Metallic Nanorod Arrays Considering Polarization Coupling

Dustin M. Lattery[1], Mingeon Kim[2], Jongin Choi[2], Bong Jae Lee[2,*], and Xiaojia Wang[1,*]

[1]Department of Mechanical Engineering, University of Minnesota, Minneapolis, MN 55455, USA
[2]Department of Mechanical Engineering, Korea Advanced Institute of Science and Technology, Daejeon 34141, South Korea
[*]Corresponding authors: bongjae.lee@kaist.ac.kr (B.J. Lee), wang4940@umm.edu (X.J. Wang)

1 Coordinate transformation of relative permittivity tensor

The Fresnel coefficients, and thus the reflectivity and transmissivity of the uniaxial crystal interface, can be obtained by solving Maxwell's equations. A coordinate transform on the local permittivity tensor is applied to convert the anisotropic permittivity from a local coordinate system defined by the geometric orientation of the NRs to the global coordinate system for the ease of tracing the light propagation (Figure 1)[1].

For uniaxial materials, the relative permittivity tensor (the permittivity tensor divided by the vacuum permittivity) is given by the diagonal matrix:

$$\bar{\varepsilon} = \begin{pmatrix} \varepsilon_{\mathrm{O}} & 0 & 0 \\ 0 & \varepsilon_{\mathrm{O}} & 0 \\ 0 & 0 & \varepsilon_{\mathrm{E}} \end{pmatrix} \tag{1}$$

Note that for the NR arrays discussed above, the shape-induced anisotropy induces a highly metallic behavior along the extraordinary direction and a dielectric behavior along the ordinary direction. This leads to a hyperbolic feature of the array-like structure (*i.e.*, $\Re(\varepsilon_{\mathrm{E}}) < 0$ and $\Re(\varepsilon_{\mathrm{O}}) > 0$) following a crossover point where the real portion of one component changes from positive to negative at a certain wavelength. This point is dependent on the material optical constants and geometry.

To transform $\bar{\varepsilon}$ into the global coordinates, it is necessary to define these two coordinate systems. The relationship between the global & local coordinate systems is shown in Figure 1b. The uniaxial behavior of this material simplifies this calculation from requiring nine angles to a transform that requires only three angles. These Euler angles are often defined as cosines of angles to simplify the notation, and for this system are $\alpha = \cos(\psi)\sin(\theta_t)$, $\beta = \sin(\psi)\sin(\theta_t)$, and $\gamma = \cos(\theta_t)$, where θ_t and ψ are the tilt angle and azimuthal angle, respectively (see Fig. 1). Applying the coordinate transform to the local, uniaxial permittivity tensor results in the 3×3 relative permittivity tensor:

$$\bar{\varepsilon} = \begin{pmatrix} \varepsilon_{\mathrm{O}} + \alpha^2(\Delta\varepsilon) & \alpha\beta(\Delta\varepsilon) & \alpha\gamma(\Delta\varepsilon) \\ \alpha\beta(\Delta\varepsilon) & \varepsilon_{\mathrm{O}} + \beta^2(\Delta\varepsilon) & \beta\gamma(\Delta\varepsilon) \\ \alpha\gamma(\Delta\varepsilon) & \beta\gamma(\Delta\varepsilon) & \varepsilon_{\mathrm{O}} + \gamma^2(\Delta\varepsilon) \end{pmatrix} = \begin{pmatrix} \varepsilon_{xx} & \varepsilon_{xy} & \varepsilon_{xz} \\ \varepsilon_{yx} & \varepsilon_{yy} & \varepsilon_{yz} \\ \varepsilon_{zx} & \varepsilon_{zy} & \varepsilon_{zz} \end{pmatrix} \tag{2}$$

where $\Delta\varepsilon = \varepsilon_{\mathrm{E}} - \varepsilon_{\mathrm{O}}$ describes the difference between the material dielectric functions in response to the primary extraordinary and ordinary waves. Using the relationships discussed in Ref.[1], this permittivity tensor can be used to calculate Fresnel coefficients, including: r_{ss} (the Fresnel coefficient corresponding to s-polarized light that is reflected as s-polarized light), r_{sp} (s-polarized light that is reflected as p-polarized light), r_{ps} (p-polarized light that is reflected as s-polarized light), and r_{pp} (p-polarized light that is reflected as p-polarized light).

Note that for isotropic cases, or cases where the optical axis is inside the plane of incidence ($\psi = 0°$ or $180°$), the cross-polarization terms (r_{sp} and r_{ps}) will reduce to zero. In these special situations, there is no coupling between electric fields for the ordinary and extraordinary cases, leading to decoupled cases. For these decoupled cases, $\beta = 0$, which causes the permittivity tensor to simplify to an admittance and impedance approach as described by Gaylord *et al.*[2], resulting in r_{ss} and r_{pp} being equivalent to r_{12s} and r_{12p} as defined by Wang *et al.*[3].

2 Calculation for the coupled Fresnel coefficient

The solution for Maxwell's equation in three-dimensional space for non-conducting, non-magnetic, and anisotropic materials can be shown as

$$\begin{pmatrix} \varepsilon_{xx} - (k_z/k_0)^2 & \varepsilon_{xy} & \varepsilon_{xz} + k_x k_z/k_0^2 \\ \varepsilon_{yx} & \varepsilon_{yy} - \frac{k_x^2+k_z^2}{k_0^2} & \varepsilon_{yz} \\ \varepsilon_{zx} + k_x k_z/k_0^2 & \varepsilon_{yz} & \varepsilon_{zz} - (k_x/k_0)^2 \end{pmatrix} \begin{pmatrix} E_x \\ E_y \\ E_z \end{pmatrix} = 0, \tag{3}$$

where $k_0 = (\omega_0/c_0)$ is the wavevector of free space, $k_x = k_0 n_1 \sin(\theta_i)$ is the wavevector in the global x-direction, n_1 is the index of refraction of the material that the incoming light is traveling through prior to the NR array, k_z is the wavevector in the z-direction, and E_i is the electric field in the x, y, or z-direction.

This solution results in eigenvalues that correspond to allowed values of k_z and eigenvector that correspond to the allowed values of electric field. In an isotropic material, there exists only one unique solution for k_z and the electric field, but for uniaxial materials, there are two allowed wavevectors, and thus, two allowed electric fields. These two solutions correspond to the ordinary and extraordinary waves discussed previously.

Ordinary wave propagation in the nanorod array can be described with the wavevector

$$k_{zo}^2 = \varepsilon_O k_0^2 - k_x^2 \tag{4}$$

with the resulting electric field vector given by

$$E_O = N_O \begin{pmatrix} -\beta k_{zo} \\ \alpha k_{zo} - \gamma k_x \\ \beta k_x \end{pmatrix} \tag{5}$$

where N_O is a normalization factor that normalizes the magnitude of the vector to unity. Note that this derivation does not require the absolute magnitudes to find the correct solutions. Mathematically, eigenvectors are normalized because any scalar multiple of the eigenvector is a correct solution, which is true here as well. The relative magnitude of each component is important, not the actual magnitude, for the determination of the Fresnel coefficients.

The other eigenvalue corresponds to extraordinary propagation, and is given by

$$k_{ze} = \frac{\sqrt{\varepsilon_O[\varepsilon_E(\varepsilon_O + \gamma^2 \Delta\varepsilon)k_0^2 - (\varepsilon_E - \beta^2 \Delta\varepsilon)k_x^2]} - \alpha\gamma\Delta\varepsilon k_x}{\varepsilon_O + \gamma^2 \Delta\varepsilon} \tag{6}$$

with an electric field of

$$E_E = N_E \begin{pmatrix} \alpha k_{zo}^2 - \gamma k_x k_{ze} \\ \beta \varepsilon_O k_0^2 \\ \gamma(\varepsilon_O k_0^2 - k_{ze}^2) - \alpha k_x k_{ze} \end{pmatrix} \tag{7}$$

These definitions can now be used to calculate the Fresnel coefficient of the nanorod surface. This can be achieved by tracking and arbitrary TE (only non-zero in the y-direction, s-polarization) or TM (only non-zero in the x and z-directions, p-polarization) polarized electric field of unit magnitude at a given θ_i. Applying continuity boundary conditions results in a linear set of four equations with four unknowns (the Fresnel coefficients in transmission and reflection) for each polarization.

$$\begin{aligned} r_{ss} &= [(k_0 n_1 \cos(\theta_i) - k_{ze}) A E_{Ey} - (k_0 n_1 \cos(\theta_i) - k_{zo}) B E_{Oy}]/C \\ r_{sp} &= [2 n_1 k_0 (A E_{Ex} - B E_{Ox}]/C \\ r_{pp} &= (2\frac{n_1 k_0}{C\cos(\theta_i)})[(n_1 k_0 \cos(\theta_i) + k_{ze}) E_{Ox} E_{Ey} - (n_1 k_0 \cos(\theta_i) + k_{zo}) E_{Ex} E_{Oy}] - 1 \\ r_{ps} &= 2 n_1 k_0 (k_{ze} - k_{zo}) E_{Oy} E_{Ey}/C \end{aligned} \tag{8}$$

A, B, and C are coefficients that appear multiple times and are defined as:

$$\begin{aligned} A &= (k_0 n_1 \cos(\theta_i) + k_x \tan(\theta_i) + k_{zo}) E_{Ox} - k_x E_{Oz} \\ B &= (k_0 n_1 \cos(\theta_i) + k_x \tan(\theta_i) + k_{ze}) E_{Ex} - k_x E_{Ez} \\ C &= (k_0 n_1 \cos(\theta_i) + k_{ze}) A E_{Ey} - (k_0 n_1 \cos(\theta_i) + k_{zo}) B E_{Oy} \end{aligned} \tag{9}$$

References

1. Lekner, J. Reflection and refraction by uniaxial crystals. *J. Physics: Condens. Matter* **3**, 6121 (1991).

2. Weis, R. S. & Gaylord, T. K. Electromagnetic transmission and reflection characteristics of anisotropic multilayered structures. *J. Opt. Soc. Am. A* **4**, 1720–1740 (1987).

3. Wang, X. J., Abell, J. L., Zhao, Y.-P. & Zhang, Z. M. Angle-resolved reflectance of obliquely aligned silver nanorods. *Appl. Opt.* **51**, 1521–1531 (2012).
